# Acceso a ligadura tubaria: análisis de las trayectorias y experiencias de las mujeres en el subsector público de salud de la ciudad de Santa Rosa, La Pampa, Argentina

**DOI:** 10.18294/sc.2025.5346

**Published:** 2025-07-04

**Authors:** Pilar Galende Villavicencio

**Affiliations:** 1 Médica, especialista en Medicina General. Maestranda en Género, Sociedad y Política. Presidenta, Federación Argentina de Medicina General. Integrante, proyecto Políticas y Acciones Colectivas, Facultad de Ciencias Humanas, Universidad Nacional de La Pampa, Santa Rosa, Argentina. pilargalende@gmail.com Universidad Nacional de La Pampa Facultad de Ciencias Humanas Universidad Nacional de La Pampa Santa Rosa Argentina pilargalende@gmail.com

**Keywords:** Derechos Reproductivos, Ligadura Tubaria, Accesibilidad a los Servicios de Salud, Género, Argentina, Reproductive Rights, Tubal Ligation, Accessibility to Health Services, Gender, Argentina

## Abstract

El acceso a ligadura tubaria se encuentra regulado en la provincia de La Pampa por la Ley Provincial 2079 y la Ley Nacional 26130 y, si bien el único requisito legal es ser mayor a 16 años y la firma de un consentimiento informado, en la práctica se evidencian barreras derivadas de una heteronormatividad institucionalizada, que privilegia algunos perfiles de mujeres, mientras se mantienen resistencias a realizarla a todas las solicitantes en tanto titulares de derecho. Este estudio se propone describir y analizar las trayectorias y experiencias de las mujeres que solicitaron una ligadura tubaria -accedieran o no a su realización- en el subsector público de salud de la ciudad de Santa Rosa, La Pampa, a través de un enfoque teórico-metodológico cualitativo basado en la teoría fundamentada. Se realizaron 18 entrevistas en profundidad durante 2022 y 2023. Los resultados muestran los efectos de la valoración social de la maternidad en los servicios de salud, que sitúa a las mujeres en posición de subordinación de su autonomía en tanto derecho a tomar decisiones sobre sus funciones reproductivas frente al poder médico.

## Abstract (en)

Tubal ligation access in the province of La Pampa is regulated by Provincial Law 2079 and National Law 26130. Although the only legal requirements are to be over 16 years of age and to sign an informed consent form, in practice there are barriers stemming from institutionalized heteronormativity. This normative framework privileges certain profiles of women while resisting the procedure for others, despite their status as rights-holders. This study aims to describe and analyze the trajectories and experiences of women who requested a tubal ligation - whether or not they ultimately obtained the procedure - in the public health subsector of the city of Santa Rosa, La Pampa. The research adopts a qualitative theoretical-methodological approach based on grounded theory. A total of 18 in-depth interviews were conducted between 2022 and 2023. The findings reveal the effects of the social valuation of motherhood within health services, which places women in a subordinate position regarding their autonomy and their right to make decisions about their reproductive functions in the face of medical authority.

## INTRODUCCIÓN

El acceso a la ligadura tubaria se inscribe en el contexto más amplio del reconocimiento de los derechos sexuales y reproductivos, en los que la provincia de La Pampa ha sido pionera: tuvo la primera ley de derechos reproductivos del país^1^ y se adelantó también a la nación en el derecho a la anticoncepción quirúrgica, cuando sancionó en 2003 la Ley 2079 sobre el Ejercicio de las Actividades de Salud^2^, que incluyó en el artículo 17 la realización de ligadura de trompas de Falopio entendida como derecho humano y, por lo tanto, responsabilidad del Estado^3^.

A nivel nacional, la sanción de la Ley 26130 de Contracepción Quirúrgica^4^, en 2006, inició un proceso de políticas públicas destinadas a garantizar el acceso, para lo cual el Ministerio de Salud elaboró una guía sobre ligaduras tubarias, publicada en 2008, en la que se normatizaron sus contenidos a fines de brindar a las y los profesionales de la salud lineamientos precisos sobre el procedimiento. Los únicos requisitos mencionados para acceder a la práctica son la firma de un consentimiento informado y la mayoría de edad, que más tarde, en 2015, según las modificaciones del Código Civil y Comercial, se estableció en los 16 años. Además, la guía explicita que, al tratarse de una decisión personal, no se requiere el consentimiento del cónyuge o pareja ni autorización judicial, excepto en las personas declaradas incapaces (lo cual fue modificado en 2021, mediante la Ley 27655, habilitando a personas con discapacidad a brindar su consentimiento). 

Sin embargo, la evidencia disponible coincide en la existencia de barreras de accesibilidad, derivadas de una heteronormatividad institucionalizada^5^, que se expresa en sesgos de género vinculados a la femineidad y la maternidad, de modo que se privilegian algunos perfiles de mujeres, en referencia a haber “cumplido” con ser madre, y tener muchos hijos e hijas^6^, a la vez que se mantienen resistencias institucionales a realizar la ligadura a todas las solicitantes, en tanto titulares de derechos^7^^).^ En este mismo sentido, según un informe del Consorcio Nacional de Derechos Reproductivos y Sexuales (CoNDeRS)^8^, del 2010, que incluyó en su monitoreo a La Pampa, estas barreras de acceso derivan de manejos de poder al interior del consultorio médico, que limitan o impiden la decisión autónoma de las mujeres, imponiendo mínimos de edades y/o cantidad de hijas e hijos como requisitos. Otro estudio realizado en la Ciudad Autónoma de Buenos Aires^9^, que analiza las demandas de la ciudadanía ante la Defensoría del Pueblo para acceder a una ligadura tubaria, reafirma la necesidad de justificación que se impone a las mujeres, fundamentalmente por padecimientos físicos preexistentes o concurrentes con un embarazo, por antecedentes obstétricos de riesgo y en menor medida por dificultades económicas que exigen limitar el tamaño de la familia.

Se parte de los referentes conceptuales de experiencias y trayectorias, entendidas como una manera singular de recorrer el espacio social, donde se expresan las disposiciones del *habitus*^10^, para comprender el acceso a la ligadura tubaria desde las perspectivas de las mujeres que deciden su realización, asumiendo para ello un enfoque relacional de género^11^. Este enfoque, aplicado a la comprensión de los fenómenos vinculados a la salud, permite entender las posiciones de subordinación social en que sitúa a las mujeres la valoración social de la maternidad. La ligadura, por tratarse de un método anticonceptivo irreversible, desafía fuertemente la heteronormatividad hegemónica, que privilegia la heterosexualidad reproductiva sobre otras formas de ejercicio de la sexualidad no reproductivas. Esta normatividad, reflejada en las instituciones de salud, se convierte en un factor decisorio en los límites y alcances del acceso a la ligadura tubaria.

Según Castro y López Gómez^12^, esta función de algún modo “pedagógica” de las y los profesionales de la salud responde a la autonomía profesional en el campo de la atención médica, en la que se articulan un saber técnico-científico y un poder cultural-moral, en la cual la capacidad de regulación externa basada en normas y consensos es casi nula. Retomando el concepto de *habitus*, el agente de la práctica se reproduce culturalmente como sujeto de su profesión, dotando de autoridad técnica apreciaciones de índole ideológico o religioso sobre las mujeres. Dichas apreciaciones funcionan en calidad de juicio moral que, enlazadas con el juicio clínico, determinan las decisiones asistenciales de cada profesional como un “deslizamiento” de un proceder de carácter técnico-científico hacia una acción de índole personal-moral.

A más 15 años de sancionada la ley, la falta de conocimiento acerca del modo en que las usuarias de los servicios públicos de salud de la provincia de La Pampa experimentan las dificultades de acceso a este derecho y el impacto en su salud integral y sus decisiones reproductivas, invisibiliza las problemáticas que enfrentan para gozar plenamente de sus derechos reproductivos. El objetivo general del estudio fue describir y analizar las trayectorias y experiencias de las mujeres que solicitaron una ligadura tubaria, accedieran o no a su realización, en el subsector público de salud de la ciudad de Santa Rosa, entre los años 2010 y 2020 (momento en el que debido al inicio de la pandemia de covid-19 se suspendieron los turnos quirúrgicos). A través de un enfoque teórico-metodológico cualitativo, se buscó recuperar las perspectivas de las mujeres en torno a la accesibilidad a este derecho, sus modos de vivenciar el proceso, así como identificar los obstáculos y/o facilitadores que experimentaron para su realización, de modo de contribuir con información al diseño de políticas públicas que garanticen el acceso.

## MATERIALES Y MÉTODOS

El estudio se llevó a cabo a través de un enfoque teórico-metodológico cualitativo, con un diseño flexible basado en la metodología de la teoría fundamentada^13^. Se tomaron como unidades de análisis a las mujeres que solicitaron una ligadura tubaria en el subsector público de salud de la ciudad de Santa Rosa, provincia de La Pampa, entre 2010 y 2020. Se incluyeron mujeres entre 18 y 45 años, accedieran o no a su realización efectiva. Se excluyeron aquellas que hicieron la solicitud fuera del período de análisis, las que accedieron a la ligadura en el subsector privado y, por sus implicancias éticas, a las mujeres que se encontraban bajo atención médica por parte de la investigadora.

El ámbito de estudio se sitúa en la ciudad de Santa Rosa, cuya red de servicios públicos está conformada por 13 centros de atención primaria de salud (que constituyen el Área Programática de Santa Rosa), el Hospital Comunitario “Evita” de atención ambulatoria y el Hospital “Dr. Lucio Molas”, único efector que cuenta con capacidad para realizar la práctica quirúrgica.

El trabajo de campo se realizó entre los meses de diciembre de 2022 y octubre de 2023. Se obtuvo un muestreo teórico, siguiendo los principios de propósito teórico y relevancia. La selección de las personas participantes fue no probabilística e intencional, hasta lograr la saturación teórica de las dimensiones del estudio. Se utilizó la técnica bola de nieve^14^ a partir de contactos con redes de mujeres. Los primeros contactos fueron generados a través de distintas organizaciones sociales y barriales, con las que existían vínculos de confianza con la investigadora, como el Desayunador de Villa Germinal, la Red de Profesionales de la Salud por el Derecho a Decidir, la Colectiva Feminista Todas Somos Andrea, y el Merendero Fuerza Joven de Villa Parque. A través de estas organizaciones se facilitaron otros nuevos contactos con mujeres de distintos puntos geográficos de la ciudad. Entre las dificultades para la participación en el estudio pueden mencionarse aspectos vinculados, fundamentalmente, a la carga de cuidados de las mujeres contactadas y, en menor medida, a cuestiones de índole laboral que implicaron una disponibilidad horaria flexible por parte de la investigadora.

Con relación al instrumento de recolección de datos, se realizaron entrevistas en profundidad para la reconstrucción de las trayectorias y el conocimiento de las experiencias de las mujeres, en tanto técnica cualitativa que propicia el diálogo con las participantes para el abordaje de su universo de significados^15^. Al inicio de la entrevista, se completó un cuestionario a fines de realizar una caracterización sociodemográfica de las mujeres. Se consideraron las siguientes variables: edad, situación de pareja, escolaridad (máximo nivel educativo alcanzado), situación laboral (empleo formal/informal-desocupación), historia reproductiva (gestas previas/abortos/partos/cesáreas) y cobertura de salud (público, obra social, seguro privado). 

Si bien la entrevista funcionó a modo de guía, se siguieron como ejes los procesos de averiguación, motivos y toma de decisión (se indagaron las razones que llevaron a la ligadura tubaria, el acceso a información, las personas que acompañaron la decisión, entre otras); el circuito médico recorrido y el rol del equipo de salud (se consultó por los sitios de consulta y las respuestas recibidas por las y los profesionales); las barreras y los facilitadores percibidos durante dicha trayectoria; y, por último, se abordó el después de la ligadura tubaria o la no ligadura, tanto en las dimensiones físicas, como sexuales, emocionales y sociales, a fines de valorar los impactos del acceso/no acceso.

Se realizaron un total de 18 entrevistas, que fueron realizadas en distintos espacios de salud pertenecientes al Área Programática de Santa Rosa, algunas en el domicilio de las mujeres y otras en el ámbito comunitario, como los merenderos y desayunadores mencionados. En todos los casos, se explicó a las mujeres las características y finalidades del estudio y se realizó la firma de un consentimiento informado. El promedio de duración de las entrevistas fue de 40 minutos. Las mismas fueron grabadas en audio con la autorización de las participantes. Tanto el proyecto como el consentimiento fueron revisados y aprobados por el Consejo Provincial de Bioética del Ministerio de Salud de La Pampa. A fines de preservar la identidad de las entrevistadas, se utilizan pseudónimos en la presentación de los resultados.

Por tratarse de un abordaje cualitativo, el procesamiento de datos fue simultáneo al trabajo de campo. Las entrevistas fueron desgrabadas, conformando el corpus empírico sobre el cual se realizó un análisis de contenido operacionalizado en los siguientes momentos: análisis individual de cada entrevista para la reconstrucción del caso (dimensión horizontal) y su codificación manual en un cuadro para la elaboración de las categorías emergentes. Para el análisis comparativo (dimensión vertical) se utilizó el método de comparación constante^13^, para lo cual se elaboraron dos matrices correspondientes al cuestionario inicial y a las categorías emergentes de las entrevistas, que permitió delimitar las trayectorias y experiencias de las mujeres en sus similitudes y singularidades y elaborar nuevas categorías y propiedades de análisis. Por último, los resultados obtenidos se fueron articulando con las referencias teóricas del estudio para el análisis final.

Dada la pertenencia de la investigadora al ámbito de estudio, el proceso demandó una reflexividad metodológica constante^16^ como conciencia sobre los efectos derivados de dicha inclusión.

## RESULTADOS

La muestra quedó conformada por un total de 18 mujeres de distintos puntos geográficos de la ciudad de Santa Rosa. Las edades oscilaron entre los 29 y 45 años, con un promedio de edad de 36 años. Salvo una de las mujeres, el resto se encontraban desocupadas o trabajando informalmente, y en dos casos recibían una pensión por madre de siete o más hijos e hijas. Solo tres mujeres se encontraban solteras al momento de la entrevista, mientras el resto se encontraban conviviendo en pareja. Con respecto al nivel educativo, la mayoría (15 mujeres) habían completado la primaria y comenzado la secundaria, pero no pudieron terminarla. Una de ellas había completado la secundaria y dos mujeres no habían completado los estudios primarios. Con relación a sus historias reproductivas, todas tenían hijos y/o hijas, entre tres gestas (dos casos) y ocho gestas (dos casos). En promedio, habían tenido entre cuatro o cinco embarazos, incluyendo partos, cesáreas y abortos. Con relación a la cobertura sanitaria, todas se atendían en el subsector público de salud y no contaban con obra social u otros seguros. 

Si bien en los relatos de las mujeres se evidencian distintos momentos de contacto con los servicios de salud a lo largo del período analizado, pueden delimitarse dos tipos de trayectorias:

1. Mujeres que realizan la solicitud en un primer o segundo embarazo y sin complicaciones médicas que afecten la gestación y transitan dificultades o barreras para el acceso a ligadura tubaria. Este grupo incluye a 12 mujeres con y sin acceso a la ligadura. Es decir, algunas de ellas iniciaron la solicitud y, después de una “carrera de obstáculos”, lograron acceder, mientras que otras, nunca pudieron hacerlo. A estas mujeres se las categorizó como trayectoria 1 (T1).
2. Mujeres en las que la multiparidad y/o la presencia de factores de riesgo para la salud preexistentes o vinculados a la gestación aseguran el acceso a la ligadura sin dificultades. A las seis mujeres incluidas en este grupo, se las categorizó como trayectoria 2 (T2).

Estas trayectorias son descriptas siguiendo las siguientes categorías de análisis:

1. Toma de la decisión
2. Circuitos de acceso
3. Acceso a información
4. Percepciones del proceso
5. Impacto del acceso/no acceso

### Toma de la decisión

Con respecto a la decisión de solicitar una ligadura tubaria, se analizaron los motivos, el rol de la pareja u otros vínculos afectivos y la influencia de las y los profesionales de la salud.

Respecto a los motivos, la principal razón por la que las mujeres refieren querer ligarse es la sobrecarga de cuidados que implica la maternidad. La falta de redes de apoyo para la crianza, las experiencias de ausencia paterna en los embarazos y las responsabilidades compartidas en la infancia configuran situaciones que son vivenciadas con pesar y que delimitan un sentimiento común de no querer volver a ser madres. Algunas expresiones que dan cuenta de ello son:

“*O sea, te lleva tiempo criar un hijo, alimentarlo, cuidarlo, todo te lleva tiempo y tenés que cuidar un bebé y tenés que cuidar a otro, es todo el tiempo. Y también el tiempo de uno, yo soy mamá 24 horas y ¿cuándo voy a pensar en mí?*”. (Marcela, 36 años, T1)

“*Era, primero, muy joven para tener tantos chicos de golpe y, segundo, porque no tenía trabajo, no tenía pareja estable, mi condición era sola, o sea en ese tiempo tenía solo un plan social imagínate*”*.* (Olga, 43 años, T1)

En muchos casos, la carga de cuidados se ve reforzada por las dificultades socioeconómicas, que implica para las mujeres limitaciones en la inserción en el mercado laboral, así como mayores gastos domésticos, tal como lo describen algunas de ellas:

“*Porque, como te digo, ya son tres, hoy en día está muy difícil todo, no se puede traer una criatura así al mundo, podes traerla para tenerla bien, yo como te digo estoy sola con ellos, y no es fácil, más que nada... ya te digo quedé sola con ellos y cuesta*”*.* (Daniela, 29 años, T1)

“*Me quiero operar porque hoy en día se te complica, tenés que tener trabajo, tenés que tener un lugar, tenés que estar bien porque para traer chicos al mundo y estar, ponele que se cague de hambre o no poder vestirlo o algo, es complicado, por eso también pensaba en eso yo*”*.* (Natalia, 29 años, T1)

Otros motivos referidos por las mujeres son las dificultades en el uso de otros métodos anticonceptivos reversibles. Las experiencias de fallas son relatadas como frecuentes y motivan a las mujeres a tomar decisiones más definitivas sobre la reproducción, para sentirse más seguras. Además, se refieren cuestiones vinculadas a la disponibilidad de los métodos en los servicios de salud, así como los mecanismos de acceso que, en algunas situaciones, interfieren en la continuidad de su uso correcto. Como se evidencia en estos relatos, las fallas no solo dependen del uso por parte de las mujeres, sino que -como en el caso de los anticonceptivos inyectables, en los que salud pública entrega según disponibilidad distintos insumos- en ocasiones derivan de factores institucionales:

“*Yo de mi hijo me quedé embarazada con las pastillas, tomaba la anticonceptiva y un día tomé mal, me tocaba una hora y me olvidé y la tomé al otro día y quedé re embarazada*”*.* (Irma, 40 años, T1)

“*Como siempre, me cuidé con inyectable y nunca me falló, ahora lo que me pasó que debo haber quedado embarazada, digo yo, porque hubo un tiempo que daban acá en salud pública la trimestral y después la de un mes* […] *en esos cambios supuse que quedé porque después que nació la nena* […] *yo siempre me cuide con la inyectable, nunca me dejé de poner y un momento me empezaron a doler los pechos y estaba embarazada*”. (Fabiana, 35 años, T1).

En cuanto al rol de las parejas y otros vínculos afectivos se observa que, en todos los casos, las mujeres relativizan opiniones y posturas de sus círculos íntimos y se afirman en la solicitud de la ligadura como una decisión propia y autónoma. En algunos casos, las parejas apoyaron su proceso, en otros disintieron y en la mayoría no tuvieron ningún rol:

“*No, nunca, yo siempre tomaba las decisiones sola porque me gustaba y me gusta ser independiente, yo digo si voy no le pido permiso a nadie, no le pido opinión a nadie, me gusta tomar las decisiones sola y en ese momento pasó eso*”. (Mercedes, 45 años, T1)

“*En ese momento mi pareja no quería, pero yo sí, pero yo ya tenía decidido… él no estaba de acuerdo, me decía que no, pero tampoco me acompañó, o sea, no se oponía, me salía con la mía porque yo mando, es mi cuerpo y es mi decisión*”. (Daniela, 29 años, T1)

Por último, acerca de la influencia de las y los profesionales, entre las mujeres T2 la decisión estuvo atravesada por la sugerencia médica frente a la aparición de complicaciones del embarazo u otros antecedentes de salud, por los cuales la indicación de ligarse es definitoria para esas mujeres. En cambio, para las mujeres T1 la influencia de las y los profesionales desalienta su decisión:

“*Ahí la doctora me dijo que me iba a tener que ligar, porque ya eran muchos embarazos, muchas cesáreas. A parte de eso era de que yo tenía problemas con la presión y bueno, la doctora me dio la posibilidad de no ligarme, pero me dijo si no te ligas ya puede ser que el otro embarazo tengas mucho riesgo de vida*”. (Juana, 36 años, T2)

“*Bueno, me quise operar y me dijeron que no porque tenía 20 años, era el primer embarazo, que lo pensara, que era muy chica, que yo capaz que si me operaba me iba a arrepentir, que capaz quería tener otro y como que me iba a costar*”. (Natalia, 29 años, T1)

### Circuitos de acceso

Sobre esta dimensión se analizaron: espacios de consulta, requisitos solicitados, respuesta de las y los profesionales ante la solicitud, obstáculos identificados y/o situaciones facilitadoras.

Con respecto a los espacios de consulta, son mencionados en primer lugar el Hospital Lucio Molas y en menor proporción el Hospital Comunitario Evita y los centros de atención primaria. Cabe señalar que, en todos los casos, independientemente del lugar, la solicitud de la ligadura se produce en el marco de la consulta de seguimiento del embarazo. Las y los profesionales intervinientes que se mencionan son principalmente de ginecología y, ocasionalmente, obstétricas y de medicina general. En ningún caso el pedido surge en el contexto de una consulta por anticoncepción u otras, sino exclusivamente en el proceso de control prenatal. 

Sobre los requisitos para el acceso, se observa que las mujeres que hicieron la solicitud en la primera parte del período analizado (hasta 2015), además de la firma de un consentimiento informado, debieron cumplimentar, en algunos casos, otros pedidos, como el apoyo expreso de la pareja y la consulta psicológica, dependiendo del lugar de consulta. Así, antes de esa fecha, se evidencia una diversidad de respuestas según el equipo de salud interviniente a pesar de la plena vigencia de los marcos legales mencionados. Entre las mujeres se manifiestan representaciones sobre esos requisitos, como parte habitual del proceso de solicitud que se hacía en el pasado, sin cuestionamientos, aunque en algunas situaciones, estos pedidos no contemplados por la ley hayan sido decisivos para el acceso:

“*Sí fuimos al psicólogo que te hacían ir, porque antes era más con la pareja, después se hizo más la decisión de las mujeres solas, que las mujeres tomamos la decisión de no tener más hijos y nos ligamos. Pero antes con el consentimiento de la pareja*”. (Carla, 30 años, T1)

“*Me habían quedado en llamar para el psicólogo, nunca me hicieron la entrevista con el psicólogo… quedamos que yo ahí mismo tenía a Fátima* y me hacían la ligadura, pero faltaba el psicólogo… *bueno yo seguía esperando y cuando me quise acordar nació y no me la hicieron*”. (Paula, 42 años, T1)

Con respecto a la firma del consentimiento, resulta relevante señalar que en la mayoría de las entrevistas surge un desconocimiento acerca del proceso de brindar consentimiento sobre la intervención, asimilando la firma como una cuestión de índole administrativa más que de asesoramiento y ejercicio de autonomía. Este punto resulta crucial ya que da cuenta de las limitaciones en la transparencia activa por parte de las y los profesionales para la toma de decisión de las mujeres.

“*Ella me mandó, ligación dijo, en el momento que yo fui a la cesárea de urgencia, ella puso la historia clínica debajo de la almohada y dijo ligación así que bueno… sí, creo que firmé algo, una planilla blanca, sí*”. (Flavia, 41 años, T2)

“*No, no, porque así nomás leyeron el coso… leyeron los papeles y bueno*”. (Irma, 40 años, T1)

Las respuestas de las y los profesionales de salud frente a la demanda de las mujeres es heterogénea y parece depender del lugar de atención y el equipo interviniente. Es decir, la multiplicidad de respuestas da cuenta de la ausencia de una protocolización en el acceso a la práctica, independientemente de que se trate de un centro de salud del primer nivel de atención o de complejidad creciente.

Sin embargo, las respuestas recibidas frente a la solicitud permiten diferenciar dos grupos: mujeres que reciben una primera respuesta negativa e inician una trayectoria T1 y mujeres que reciben una respuesta afirmativa que se corresponden a la trayectoria T2.

Los motivos de rechazo más referidos por las mujeres T1 se vinculan a la edad y el número de hijos y/o hijas que operan como principales obstáculos frente a la solicitud. A diferencia de los requisitos mencionados, donde se observa un cambio con los años en la respuesta de los equipos de salud, los obstáculos referidos persisten a lo largo de todo el período analizado:

“*Cuando tuve mi primer hijo que tiene una discapacidad, bueno ahí pregunté si podía hacerme la ligadura y se me negó… me dijeron que no porque era muy joven, yo por el caso de la discapacidad de mi nene pedí la ligadura por el caso que se volviera a repetir la historia*”. (Marcela, 36 años, T1)

“*Le dije que me quería ligar y ‘no porque sos joven, después vas a volver a tener una pareja y vas a querer tener un hijo...’ ahí sí me sentí medio mal, como que me cuestionaban que yo me quería ligar y ellos no querían... con la enfermera que te tomaba la presión y le decía c*ó*mo puedo hacer para ligarme, ‘preguntale al médico, pero no creo que te lo hagan porque sos muy jovencita todavía*’”. (Mercedes, 45 años, T1)

Dentro del primer grupo de mujeres que inician una trayectoria T1, puede identificarse a su vez un subgrupo de seis mujeres categorizadas como “*mujeres en lista de espera*”. En este subgrupo, las solicitantes refieren ser incorporadas en un listado de pacientes dentro del servicio de Tocoginecología del Hospital Molas que aguardan turno para la cirugía. En estos casos, las mujeres alegan largos períodos de espera (en promedio 5 años, en dos casos anotadas hace 10 años sin haber accedido hasta la fecha de la entrevista). La respuesta de las y los profesionales de salud resulta poco clara con relación a los mecanismos institucionales de seguimiento de sus solicitudes y las vías de contacto para continuar con el proceso. Los testimonios de estas entrevistadas dan cuenta de las dificultades que atraviesan para el acceso:

“*Yo fui a pedirla y me atendía una asistente social y lo único que me dijeron es que hay mucha demanda, que en algún momento te van a llamar... nunca me llamaron*”. (Sonia, 45 años, T1)

“*En la primera ventanilla, ahí, yo le pregunté al chico, le di el documento y le dije si tenía un turno para ligamiento, me agarró el documento y me dijo que estaba en lista de espera, que cualquier cosa deje el número de teléfono y me iban a llamar*”. (Irma, 40 años, T1).

Con respecto al segundo grupo de mujeres que iniciaron una trayectoria T2, se identifican como facilitadores la presencia de riesgo para la salud en el embarazo, sumado en algunos casos a la multiparidad y, en particular, las cesáreas múltiples, que promueven a médicos y médicas a sugerir la indicación de ligadura a fines de evitar posibles complicaciones futuras:

“*Sí ella me dijo, me dijo nosotras ya firmamos los papeles acá, ni bien te hacen la cesárea ya te ligan y ya después listo… firmé ahí directamente, me lo hizo firmar la doctora, porque como era de alto riesgo no me pidieron ni ir al psicólogo, nada*”. (Juana, 36 años, T2)

### Acceso a información

Sobre el acceso a la información, se analizan las fuentes a las que recurren las mujeres, su calidad y disponibilidad. Con respecto a las fuentes, las entrevistadas refieren conocer el método como resultado de las conversaciones cotidianas entre mujeres, que involucran los cuidados reproductivos, el embarazo y la crianza. En cuanto al conocimiento acerca del procedimiento quirúrgico, todas las mujeres mencionan la consulta médica como ámbito de información. Una sola entrevistada mencionó la búsqueda en Internet como modo de informarse. 

Sin embargo, las respuestas acerca de la calidad, entendida como información comprensible, completa y oportuna, difieren entre las entrevistadas. Entre las mujeres T1, se evidencia desconocimiento acerca del procedimiento e ideas confusas acerca de lo que efectivamente sucede en el cuerpo con la ligadura. La falta de explicación sobre las técnicas utilizadas aparece en todos los relatos y se traduce en incertidumbre, dado que no existen modos de comprobar su realización efectiva y se expresa en la asimilación dudosa de terminologías como “me cortaron”, “me quemaron”, “me ataron”. El proceso de asesoramiento resulta insuficiente para la mayoría de ellas, lo cual se vincula con el otorgamiento del consentimiento como un aspecto formal a cumplimentar más que de intercambio con las y los profesionales:

“*Lo que no entendí después que nunca lo consulté tampoco si fue ligadura o atadura, eso es lo que no sé, lo único que te puedo decir es que ya hacen 11 años del último embarazo y no volví a quedar*”. (Olga, 43 años, T1)

“*No, nunca, solamente me dijeron ‘te hacemos esto y ya no vas a poder tener más hijos…’ yo necesitaba una charla más profunda, saber ponele cómo iba a quedar después, una nunca sabe, porque una tiene dudas, por ahí no sabes lo que te van a hacer adentro y vos est*ás *dormida*”. (Mercedes, 45 años, T1)

Resulta relevante señalar que, entre las mujeres T2, la información provista por el equipo de salud parece más clara para las mujeres, la cual es referida como satisfactoria con relación a sus inquietudes:

“*Sí ella me explicó bien me hablo de qué se trataba la ligadura, me explicó también que una vez que estás ligada no te podés desligar, hay muchas mamás que piensan que están ligadas hoy y después se pueden deshacer de la ligadura y eso no es así*”. (Juana, 36 años, T2)

“*A mí se ligaron, se cortaron y se quemaron, directamente fuiste. La mía me la hicieron completita*”. (Lucía, 38 años, T2)

Por último, sobre la disponibilidad de información, no se distinguen diferencias entre ambos grupos y la referencia es negativa en cuanto a la difusión del derecho y de la existencia de una ley que habilita a las mujeres a solicitar la ligadura sin las condiciones que les fueron impuestas en los servicios de salud:

“*Estaría muy bueno que uno entre a una salita o un hospital y haya un cartel que diga es ley la ligadura de trompas, más de una mamá lo piensa*”. (Sonia, 45 años, T1)

“*Falta mucha información, así como está la ley del aborto estaría bueno que se marque más esto, que se pueden ligar y ahorrarse un proceso que las deje marcadas, es feo*”. (Paula, 42 años, T1)

### Impacto del acceso/no acceso

Con relación al impacto, se analizan las repercusiones tanto a nivel físico como social y emocional, así como la emergencia de situaciones vinculadas al acceso y al no acceso.

Entre las mujeres T1 -que lograron ligarse- y las mujeres T2, las expresiones de alivio y tranquilidad develan la satisfacción por haber ejercido su derecho y en ningún caso se manifiesta arrepentimiento por la decisión tomada, uno de los aspectos que las y los profesionales aducen para negar su realización a las mujeres. La posibilidad de reconfigurar un proyecto de vida personal más allá de la maternidad es vivenciada como algo positivo tanto a nivel físico como social y emocional:

“*¡Por fin! Respiré y dije ayy ¡ahora sí! como venía pidiendo no quería tener más hijos ya estaba muy decidida que no quería volver a ser mamá*”*.* (Marcela, 36 años, T1)

“*Reencontrarse…como mujer tengo que volver a conocerme, la autoestima, nací mujer y ¿no me puedo realizar en otras cosas?*” (Patricia, 32 años, T1)

“*Sí cambió, una que ya pude trabajar, que no estaba con la panza y cambiaron muchas cosas porque yo trabajaba hasta con la panza*”. (Mercedes, 45 años, T1)

En cambio, las implicancias negativas sobre la salud física y mental del no acceso son relatadas con pesar por no haber accedido al procedimiento cuando fue solicitado, lo cual condujo a estas mujeres a encontrarse con embarazos no planificados y distintas consecuencias vinculadas al aborto (en dos casos abortos tardíos), a la maternidad no deseada y a los trastornos depresivos, como se muestra en estos relatos:

“*Cuando me enteré ya estaba de cuatro meses...me agarró como una depresión, no de no querer al bebé, sino de todo lo que me había pasado… me costó entender que iba a volver a tener un bebé, que iba a hacer todo lo mismo, me costó muchísimo, no es que no lo quiera a mi hijo, pero tenemos que ser realistas*”. (Carla, 30 años, T1)

“*Yo estuve en salud mental, sabía que no quería tener otro hijo porque no iba a salir más de ahí… y salí dopada, de hecho, si me preguntas no sé en qué momento quedé embarazada, ¿entendés? Entonces siento que decidieron por mí, y amo a mi hija, pero no era lo que yo decidí en ese momento, mi pensamiento era otro, yo estaba averiguando para no tener más hijos y quedé embarazada*”. (Patricia, 32 años, T1)

### Percepciones del proceso

En esta última dimensión se analiza cómo fue percibida la respuesta de las y los profesionales de salud, el modo en que las mujeres fueron experimentando procesos de fortalecimiento o no en cuanto a su solicitud y la forma en que significan estas vivencias. 

Con respecto a las mujeres T1, todas las entrevistadas experimentan sentimientos de vulneración y atropello a sus decisiones y sus expresiones dan cuenta de las asimetrías de poder que operan en torno al ejercicio de decisiones sobre el propio cuerpo de las mujeres. Las vivencias que transitan frente a la palabra del médico o de la médica son de sumisión e impotencia por no sentirse contempladas en sus decisiones:

“*Yo en ese momento no sentí que tuvieran en cuenta lo que me estaba pasando... de lo que yo quería... o sea no fui escuchada... lo tomaron como un pedido al pasar, como de otra persona y para* mí tenía mucho significado no volver a tener otro *hijo, pero bueno, en ese momento no fui escuchada*”. (Marcela, 36 años, T1)

“*Y mucho no hacés, porque ellos son la autoridad, mandan ellos y una no puede hacer nada… porque lamentablemente es así, no podés pasar sobre ellos, ellos son los que te dan el sí o te dan el no...mucho no podés hacer*”. (Daniela, 29 años, T1)

Una mención aparte, se realiza sobre las “mujeres en lista de espera”, quienes además de la percepción de vulneración, refieren experimentar falta de respuestas concretas por parte de los equipos que, en muchos casos, terminan por desalentarlas en su solicitud:

“*Cuando yo la pedí, la verdad, siento que quedo ahí archivada en un cajón… y siendo que yo fui varias veces, o sea, ponele unas dos veces al año a preguntar... che, yo pedí para la ligadura y… ‘ya te van a llamar, hay mucha demanda’ …iba al hospital, ahí a la ventanilla, y me mandaban a la parte de las embarazadas… ahí por ventanilla me atendía una chica... yo le decía estoy anotada por una ligadura de trompas y me dijo ‘ya te van a llamar’… sentía que iba a pedir, no sé... un pedazo de pan... es como que vos ibas a pedir algo como con miedo… entonces fui dos o tres veces más y no fui más*”. (Sonia, 45 años, T1)

“*Y yo… uno se siente como olvidado como mujer, si a mí me hubiesen ligado yo no hubiese pasado el proceso que pasé con el bebé y me hubiese ahorrado un montón de angustia porque eso también me marcó mucho... después ya te digo me quedaron en llamar y ya no… perdí la esperanza*”. (Paula, 42 años, T1)

En cambio, las mujeres T2 manifiestan haberse sentido acompañadas por el equipo de salud y refieren satisfacción con respecto al proceso transitado:

“*Ya cuando fui a cesárea estaba todo listo para hacérmela… ningún problema, ella a mí me dijo ‘vamos a hacer una charla primero y, si vos decís que sí bárbaro’, me atendió re bien, jamás me lo negó, ni un problema… un amor, un buen trabajo hicieron, a pesar de que yo fui muy cagona, muy bueno el trabajo*”. (Lucía, 38 años, T2)

## DISCUSIÓN

Las tensiones entre el ejercicio de autonomía sobre el cuerpo y la sexualidad por parte de las mujeres y la imposición de valores culturales-morales por parte de profesionales de salud que debieran ser garantes de derechos, se abren a otros debates posibles y necesarios.

Autores como Gonçalves Rodrigues *et al.*^17^ abordan los rechazos que reciben las mujeres de Manaus, Brasil, al solicitar la esterilización e introducen la dimensión biopolítica del poder del Estado que supone la invalidación del consentimiento de las mujeres a tomar decisiones sobre sus cuerpos, que emerge como un campo de disputa simbólica en el control de la sexualidad de las mujeres y que puede ser pensado en términos de violencia.

En ese sentido, estas dinámicas de negación del derecho, la dilación de su efectivización así como las experiencias emocionales y afectivas que transitan las mujeres en los espacios institucionales configuran situaciones de violencia contra la libertad reproductiva, según consta en el artículo 6° de la Ley Nacional 26485 como:

“…toda acción u omisión proveniente del personal de instituciones públicas o privadas de atención de la salud […] que vulnere el derecho de las mujeres a decidir libre y responsablemente si desea o no tener hijos, el número de embarazos o el intervalo entre los nacimientos”.^18^

Al respecto, cabe señalar que existen antecedentes de denuncias colectivas en nuestro país^19^, en las que estas acciones de tutelaje sobre la voluntad de las mujeres a realizar una ligadura tubaria han sido interpretados desde una dimensión jurídico-legal como violencia institucional, reconociendo que el acceso a los servicios de salud sexual y reproductiva constituye un derecho humano a garantizar por el Estado argentino sobre la base de sus compromisos nacionales e internacionales.

Desde una perspectiva interseccional^20^, surge además la necesidad de nombrar dichas experiencias en clave de discriminación, ya que el perfil de usuarias de los servicios de salud pública de gestión estatal se encuentra entre los estratos más vulnerados de nuestra sociedad, configurando de ese modo situaciones de opresión/privilegio en el acceso a la ligadura tubaria con respecto a aquellas mujeres que cuentan con seguros privados de salud o recursos económicos que permitan financiarlo. La incorporación de nuevas variables de análisis como la condición étnico/racial y la situación geográfica en una provincia con diversidad cultural y de significativa territorialidad rural, como es La Pampa, sería de vital importancia hacia investigaciones futuras en la temática.

## CONCLUSIONES

Las trayectorias que transitan las mujeres se corresponden con estudios previos en los que aquellas mujeres con problemas de salud preexistentes o vinculados a la gestación y/o multiparidad son alentadas por las y los profesionales de salud a ligarse y acceden a realizar el procedimiento sin mayores dificultades. Sus experiencias son satisfactorias y muestran conformidad con la atención recibida. En cambio, aquellas que no reúnen dichos criterios, se encuentran con diversas barreras que configuran experiencias de vulneración de sus decisiones, derivadas de una valoración de la función reproductiva de las mujeres por parte de profesionales de salud, en la que la edad y el número de gestaciones resultan determinantes. Estos “deslizamientos” en los que las y los profesionales responden más a sus visiones culturales-morales que a su saber técnico científico, conllevan un impacto en la salud de las mujeres. La invisibilidad de tales consecuencias resulta en sí misma un obstáculo a vencer a fines de contribuir en la planificación de una política pública que responda a los derechos reproductivos consagrados por ley.

A pesar de las diferencias entre ambos grupos de mujeres, el poder médico es determinante para el acceso a ligadura tubaria, tanto cuando se indica el procedimiento como cuando se lo niega o se lo obstaculiza. La sumisión de las mujeres a estas relaciones jerárquicas está atravesada, entre otros factores, por la falta de conocimiento de la ley y la apropiación de su derecho a decidir que les permita mayor exigibilidad hacia los equipos de salud. 

Además, la multiplicidad de respuestas institucionales frente a la solicitud da cuenta de la ausencia de circuitos claros que orienten a las mujeres sobre cómo proceder para acceder a ligarse. Entre las “mujeres en lista de espera” se observa, además, el desaliento frente a la falta de respuestas oportunas, que derivan en embarazos no planificados con gran repercusión física y sobre todo emocional para las mujeres. 

En síntesis, puede concluirse que los obstáculos que enfrentan las mujeres para el acceso a ligadura tubaria reflejan una tensión entre la autonomía profesional y la autonomía física de las mujeres en tanto derecho a tomar decisiones libres e informadas acerca de su cuerpo y sus funciones reproductivas, que conlleva un impacto negativo para su salud integral.

Si bien, como se ha visto, la sola existencia de las leyes no es suficiente para regular el accionar de las y los profesionales de la salud, la protocolización local de sus contenidos, así como la sensibilización y capacitación de los equipos intervinientes parece un paso necesario para reducir la variabilidad de respuestas brindadas por médicos y médicas, además de respaldar a profesionales como efectores de una política pública. El proceso de brindar consentimiento se configura, en ese sentido, como una oportunidad en salud que puede fortalecer la transparencia activa de los equipos, así como constituirse en un momento privilegiado de interacción profesional-persona usuaria, donde la autonomía profesional se vea limitada o enmarcada por la demanda de autonomía de las usuarias.

Por último, la difusión de la Ley 26130 resulta estratégica para facilitar el acceso a información sobre el derecho a la ligadura en los servicios de salud. Es de destacar que, en las valoraciones de las entrevistadas, la menor visibilidad de la oferta de métodos quirúrgicos de anticoncepción en relación con el aborto legal se constituye como una contradicción, ya que acceder en forma oportuna a una ligadura tubaria evitaría tener que tomar la decisión posterior de interrumpir un embarazo. 

En ese sentido, la disponibilidad de información segura y confiable por fuera de la consulta de control prenatal amplía las oportunidades para que las mujeres conozcan sus derechos y puedan exigir por su cumplimiento. Una política pública que aborde el acceso a una ligadura tubaria, como un tema de justicia reproductiva, debiera asumir que la decisión de tener o no tener hijos e hijas trasciende la libertad individual para elegir, y que dichas elecciones son parte de una cuestión social y de responsabilidad estatal.
